# Bibliometric Analysis of Research Papers on Academic Stress (1989-2023)

**DOI:** 10.7759/cureus.55536

**Published:** 2024-03-04

**Authors:** Namrata Dagli, Rahnuma Ahmad, Mainul Haque, Santosh Kumar

**Affiliations:** 1 Karnavati Scientific Research Center (KSRC), Karnavati School of Dentistry, Karnavati University, Gandhinagar, IND; 2 Physiology, Medical College for Women and Hospital, Dhaka, BGD; 3 Department of Pharmacology and Therapeutics, National Defence University of Malaysia, Kuala Lumpur, MYS; 4 Department of Periodontology and Implantology, Karnavati School of Dentistry, Karnavati University, Gandhinagar, IND

**Keywords:** stress, psychological stress, thematic analysis, citation analysis, overlay visualisation, mental health, network visualisation, academic stress, network analysis, bibliometric

## Abstract

This extensive study provides a comprehensive overview of the contemporary research landscape about academic stress, emphasizing on identifying the most relevant contributors and understanding prevalent trends. The analysis included 5,375 results from the PubMed database and revealed a consistent upward trajectory with fluctuations in research paper publications over the years. Network analysis and visualization were performed using the Biblioshiny app and VOSviewer software. The analysis identified that the University of Oslo has published the highest number of research papers related to academic stress. In contrast, the Netherlands, the USA, and Australia demonstrated the highest frequency of collaboration. Analysis of keywords and their co-occurrence provides an overview of the research focus and the areas associated with psychological stress due to academics. Thematic evaluation and topic trend analysis provided insights into the evolving nature of research in academic stress. The thematic map depicts two categories of themes - motor themes, including psychological stress, its epidemiology, the influence of the COVID-19 pandemic, and the mental health of university students, particularly those in medical programs; and emerging themes, including oxidative stress and risk factors, indicated evolving areas of interest. A notable observation was the scarcity of research on primary school students, signaling a gap in the existing academic stress literature. Citation analysis identified the most cited authors, countries, universities, and sources. This multifaceted examination provides a nuanced understanding of academic stress research's current state and dynamics, offering valuable insights into trends, collaborations, and thematic shifts that will guide future research in this critical field.

## Introduction and background

Academic stress, a phenomenon characterized by the overwhelming pressure and anxiety experienced due to the demands of academic life, has become a significant concern globally. Academic stress affects individuals' mental and physical well-being, academic performance, and career trajectories [1-3].

In light of the growing recognition of the detrimental effects of academic stress, scholars and researchers have devoted considerable attention to understanding its underlying causes, manifestations, and potential coping mechanisms [4-7]. This has led to a substantial body of research literature exploring various aspects of academic stress, including its psychological, social, and environmental determinants. A study done on academic stress suggests that it is positively associated with suicidal ideation. Still, this relationship is mitigated by greater resilience and adaptive coping strategies, while maladaptive coping exacerbates suicidal ideation among students [8]. Another study found a positive correlation between symptoms of stress and class participation, obligatory homework, and test-taking. Additionally, it highlighted the predictive relationship between specific stressors, age, and gender, emphasizing the need for in-depth analysis of academic stressors for various types of students so that targeted interventions can be planned accordingly to mitigate the impact of stress on students' academic performance [9].

This analysis uses bibliometric tools to identify the most relevant authors, research institutes, prevailing research trends, and thematic clusters within the academic stress literature. This bibliometric analysis aims to delve into the extensive corpus of academic stress-related literature to uncover patterns, trends, leading contributors, and key themes. By systematically examining scholarly publications, this analysis will shed light on the evolution of academic stress research and provide an overview of the intellectual landscape of academic stress, ultimately fostering a healthier and more supportive educational environment for students and scholars alike.

## Review

Materials and methods

On October 25, 2023, an electronic search was conducted on the PubMed database to identify research papers addressing academic stress. The search employed the strategy - (academic) AND (psychological) AND (stress). Various article types, including review articles, clinical trials, meta-analyses, and book chapters, were encompassed in the search. No filters were applied based on species, language, gender, journal, age, or publication date. The study selection process adhered to the Preferred Reporting Items for Systematic Reviews and Meta-Analyses (PRISMA) guidelines [10], and a flow chart was generated to illustrate this process.

The gathered data were exported to text files for subsequent analysis. For network analysis and visualization, Biblioshiny, a web-based app for comprehensive science mapping analysis [11], was utilized. Citation analysis was performed on data obtained from the Dimension database using the same search terms employed in the PubMed database. Overlay visualization was carried out using VOSviewer (version 1.6.20; https://www.vosviewer.com/) [12].

Examining research papers on academic stress involved a comprehensive analysis across various dimensions. Firstly, the study delved into publishing trends of research papers. Secondly, an exploration of the most contributing authors and leading universities in the field was conducted, shedding light on the critical contributors to academic stress research and the institutions at the forefront of this scholarly pursuit. The third dimension involved identifying the most frequently used keywords. Furthermore, the thematic evolution of academic stress research was scrutinized, seeking to understand how the focus and emphasis within this domain have evolved. Lastly, a comprehensive evaluation was undertaken to determine the most cited authors, sources, universities, and countries in the academic stress literature.

Results

**Figure 1:**
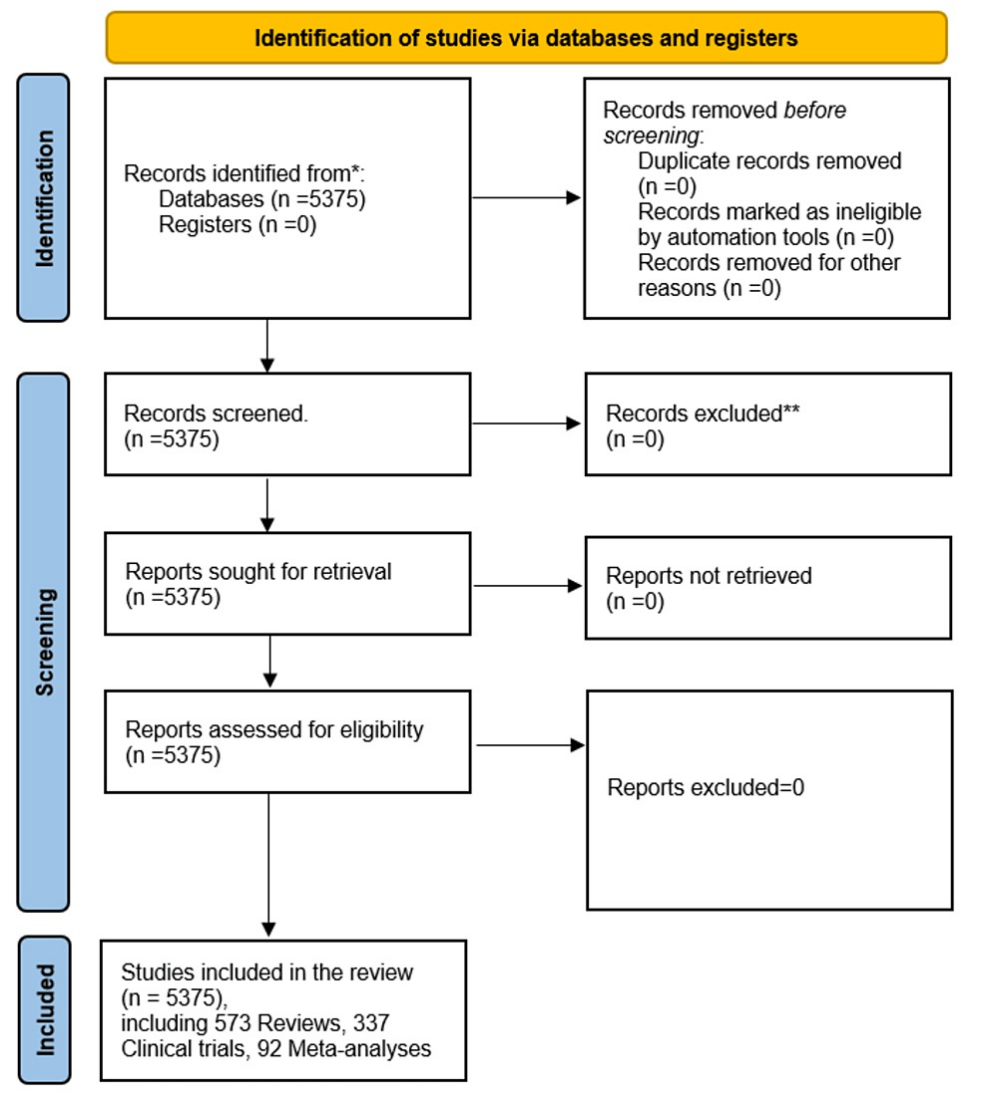
A Flow Chart Illustrating the Selection Process of Studies in the PubMed Database. Image Credit: Namrata Dagli

A total of 5,375 results appeared from an online search in the PubMed database, among which, 337 were clinical trials, 573 were reviews and systematic reviews, 92 were meta-analyses, and four were books and documents (Figure 1). The selected articles are published between 1989 and 2023.

Bibliometric analysis of the data obtained from the Pubmed database

Publishing Trend of Research Papers on Academic Stress

**Figure 2:**
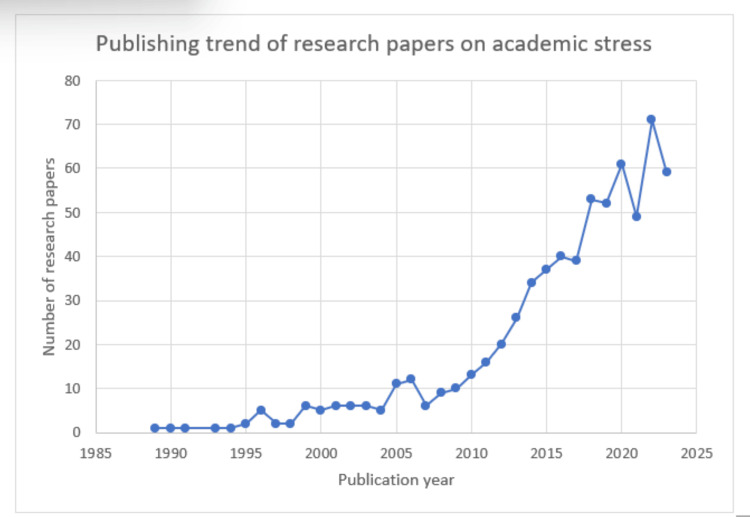
The Publishing Trend of Research Papers on Academic Stress. Image Credit: Namrata Dagli

The graph (Figure 2) illustrates a consistent upward trajectory in the number of research papers published over the years, punctuated by notable declines in 1997-1998, 2007, 2017, 2019, and 2021. This decrease was succeeded by a substantial upturn, particularly in 2021-2022, indicating a pattern of fluctuations in research paper output. The most significant drop occurred between 2020 and 2021, followed by a remarkable spike in the subsequent year.

Most Relevant Authors

**Figure 3:**
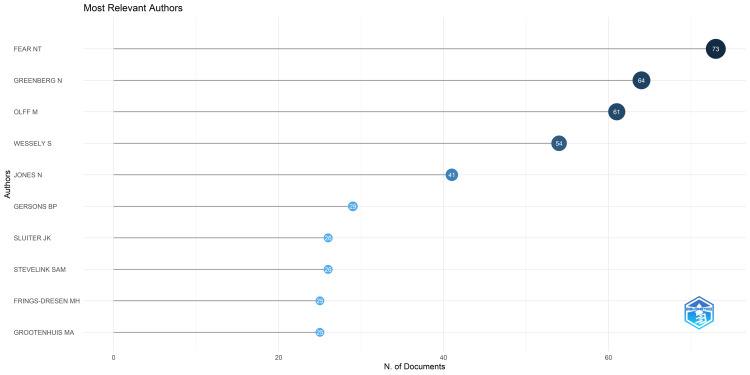
Most Relevant Authors in Research on Academic Stress. Image Credit: Namrata Dagli

Figure 3 displays the most relevant authors based on the number of published papers related to the research on academic stress. Notably, in academic stress, Fear NT, Greenberg N, and Olff M emerged as the most relevant three significant contributors, having authored 73, 64, and 61 papers, respectively. Among the 10 most influential authors highlighted, their collective work encompasses 424 research papers. Remarkably, the trio of Fear NT, Greenberg N, and Olff M accounts for a substantial portion, specifically 198 papers, constituting 46.69% of the total research output by the 10 most relevant authors in this domain.

Most Relevant Universities

**Figure 4:**
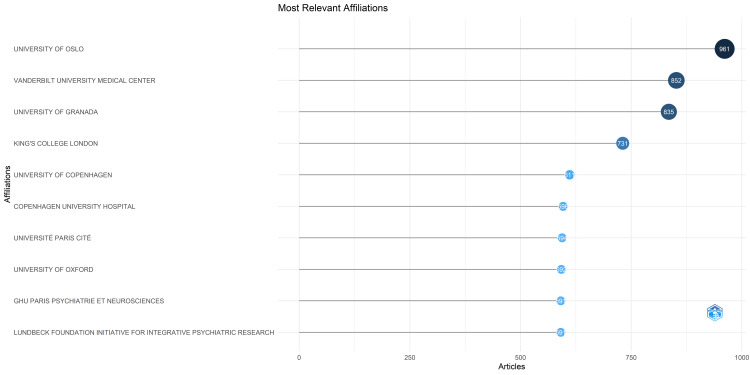
Most Relevant Universities in Research on Academic Stress. Image Credit: Namrata Dagli

Figure 4 displays the most relevant universities based on the number of published papers related to the research on academic stress. The University of Oslo emerges as a prominent hub, boasting a substantial body of work with 961 publications. Following closely behind is Vanderbilt University Medical Center, with an impressive count of 852 publications, and the University of Granada, which has contributed significantly with 835 publications. These three esteemed institutions have collectively generated 38.07% of the papers published among the 10 most relevant universities in this field, amounting to a substantial corpus of 6,954 publications (Figure 4).

Keyword Analysis

**Figure 5:**
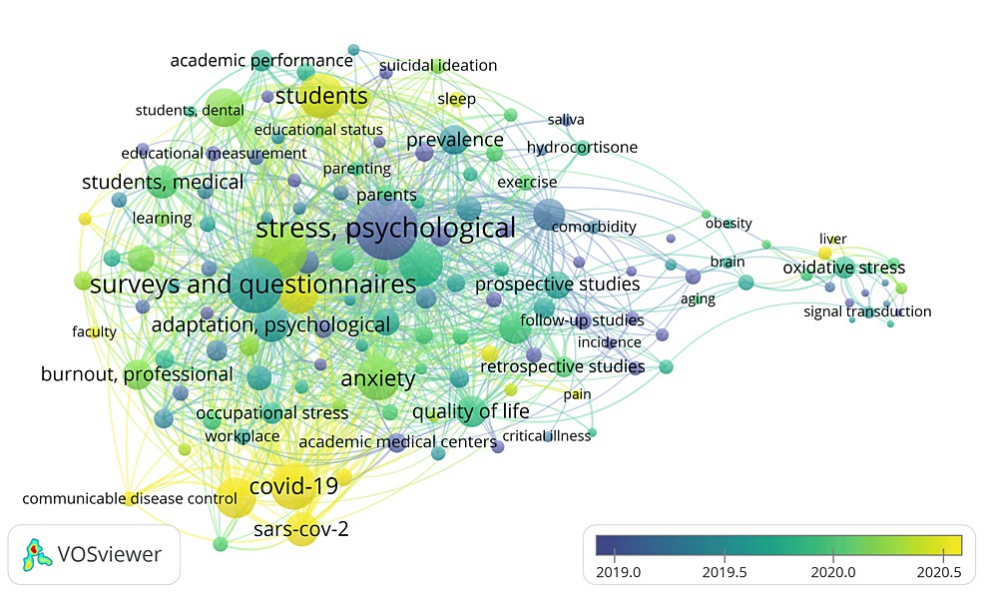
Overlay Visualization of the Co-occurrence of Keywords in Research Papers on Academic Stress. The size of the circle represents the frequency of occurrence for keywords. Lines represent the links between the items (weight-total link strength, scores-average publication year). Image Credit: Namrata Dagli

The analysis identified a total of 7,526 Medical Subject Heading (MeSH) keywords, with 1,994 keywords having a minimum occurrence of five, 502 repeating a minimum of 20 times, and 183 repeating at least 50 times. For each of these 183 MeSH keywords, the total strength of co-occurrence links with other keywords was calculated by VOSviewer software. The keywords with the greatest total link strength (TLS) are displayed in Figure 5. After removing the age and gender-related words, the analysis revealed 144 keywords interlinked with one another, forming five distinct clusters, totaling 5,989 links with a TLS of 48,627.

Notably, the prevalent keywords indicate that a significant portion of academic stress studies revolved around anxiety, quality of life, the impact of the COVID-19 pandemic, and the prevalence of stress in medical and university students. The analysis reveals the links between various keywords. Suicidal ideation is found to be linked with psychological stress and anxiety in university students. Psychological stress is linked with depressive disorder, anxiety disorder, sleep-wake disorder, and obesity. Psychological stress is also connected with oxidative stress, which is linked with neoplasm. Hydrocortisone is linked with biomarkers, stress, anxiety, saliva, and prospective studies.

The other disorders frequently studied with psychological stress are post-traumatic stress disorders and occupational stress. The factors associated with academic stress are also displayed in Figure 5, such as academic performance, educational status, self-concept, psychological adaptation, and psychological resilience.

In the graphical representation, the yellow section signifies recent research endeavors about academic stress, centering on the mental health of students, the impact of the ongoing COVID-19 pandemic, anxiety disorders, pain, infectious disease control, and psychological burnout (Figure 5).

Trend Topic Analysis and Thematic Map

In the realm of academic stress research spanning the period from 2001 to 2010, investigations primarily delved into areas such as work schedule tolerance, personality assessment, divorce-related stressors, problem-solving strategies, gender identity considerations, internal-external locus of control, relaxation therapy efficacy, social adjustment challenges, workload management, achievements, educational metrics, social environment dynamics, and significant life events.

Subsequently, between 2010 and 2020, the thematic landscape evolved to encompass a more nuanced exploration, featuring topics such as psychological testing methodologies, severity of illness indices, self-concept evaluations, job satisfaction assessments, gender-specific stress factors, analyses of educational status implications, socioeconomic determinants, social support structures, quality-of-life assessments, school-related stressors, personal satisfaction inquiries, mental health investigations, and mindfulness practices. In the period spanning 2019 to 2021, the research focus notably shifted due to the unprecedented challenges posed by the COVID-19 pandemic. The most recent trend identified in contemporary studies revolves around the concept of internet addiction disorder.

**Figure 6:**
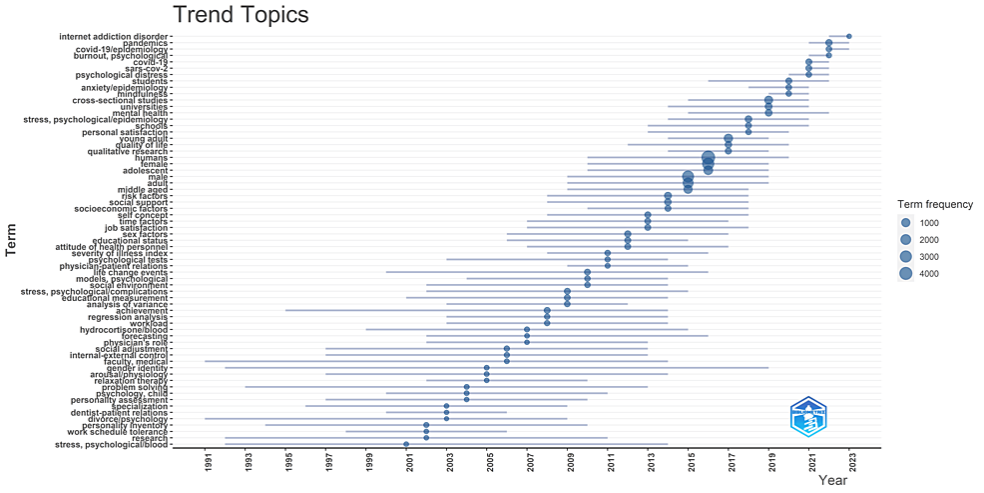
Trend Topic Analysis of Research Papers on Academic Stress. Image Credit: Namrata Dagli

This analysis underscores the dynamic shifts and evolving themes within academic stress research, reflecting the field's responsiveness to the changing socio-cultural and technological landscape (Figure 6).

**Figure 7:**
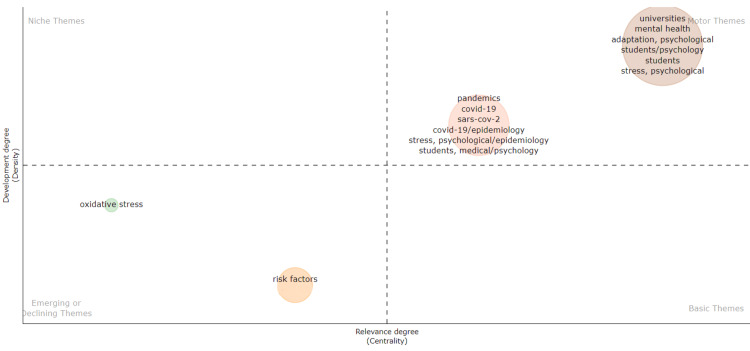
Thematic Map of Themes on Academic Stress Research. Image Credit: Namrata Dagli

A thematic map is a visual representation of the thematic structure of a research field. It is divided into four categories: motor themes, basic themes, niche themes, and emerging or declining themes. Motor themes, representing pivotal research areas steering the field, include two clusters. The more considerable cluster consists of the more relevant and developed themes - university, students, psychological adaptation, psychological stress, and mental health. The small cluster contains less developed and less relevant themes than the big cluster, such as the COVID-19 pandemic, SARS-CoV-2, epidemiology of COVID-19 and psychological stress, and psychology of the medical students. Oxidative stress and risk factors are emerging themes progressively gaining significance within the field yet to achieve mainstream recognition. No basic themes and niche themes are displayed in the thematic map (Figure 7).

Thematic Evolution Analysis

**Figure 8:**
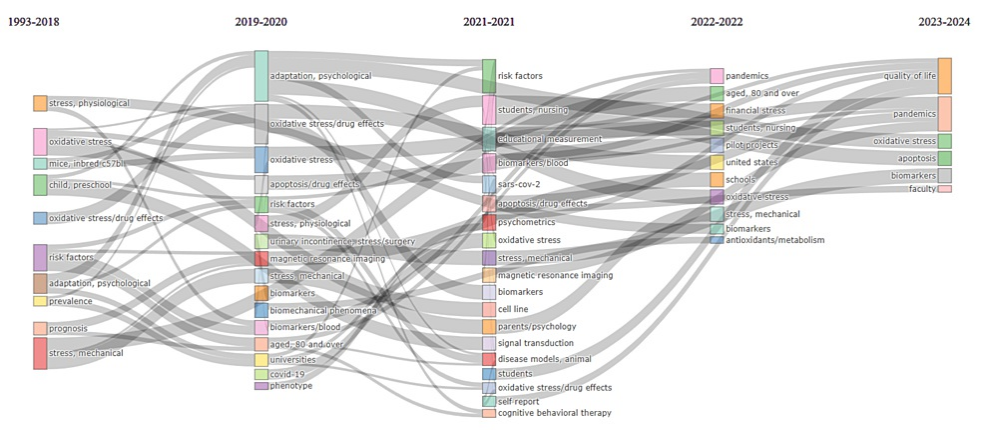
Thematic Evolution Analysis (by Biblioshiny). It Identifies Evolution in Study Themes Related to Research on Academic Stress. Image Credit: Namrata Dagli

The analysis of thematic evolution presented in Figure 8 reveals a predominant emphasis on research concerning preschool children, psychological adaptation, and oxidative stress before 2018. Between 2019 and 2020, the research shifted its focus to psychological adaptation, university students, and COVID-19. In 2021-2022, attention turned to nursing students, psychometrics, cognitive behavior therapy, biomarkers, and the pandemic. By 2023, the research focused on biomarkers, oxidative stress, apoptosis, the pandemic, and quality of life. This study suggests a transition in the population targeted for academic stress research, moving from preschool children to university and nursing students. After 2019, there was a concentrated focus on the COVID-19 pandemic and biomarkers, with oxidative stress consistently being a central theme. Furthermore, a noticeable shift is observed from examining prevalence and prognosis to psychometric assessment and, ultimately, evaluating quality of life.

Collaboration Analysis of Corresponding Authors' Countries

**Figure 9:**
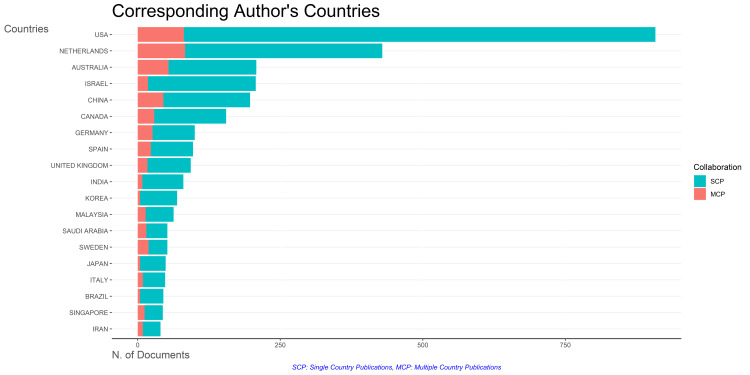
Analysis of Countries and Their Collaboration Frequency. Image Credit: Namrata Dagli

Figure 9 reveals that most academic stress research papers, regardless of origin, are single-country publications. Multiple-country publications are notably scarce, with prominent collaborative efforts observed among authors from the Netherlands, followed closely by those from the USA and Australia.

Citation Analysis of the Data Obtained From the Dimension Database

**Figure 10:**
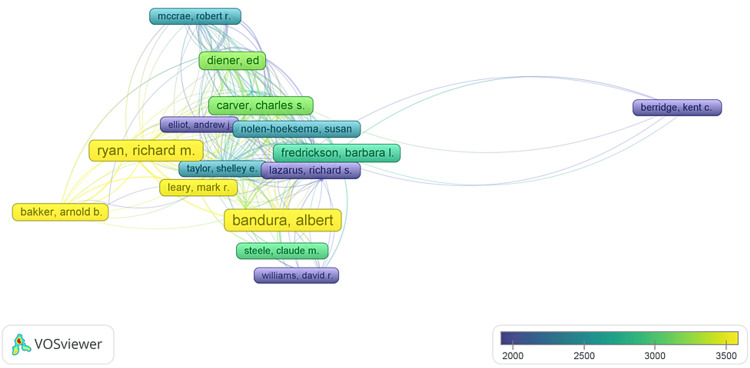
Overlay Visualization of the Most Cited Authors. The size of frames and fonts represents the number of citations (weight-citation). Lines represent the links between the items. The color of an item represents the average citation of the authors and ranges from blue (lowest score) to green to yellow (highest score). Image Credit: Namrata Dagli

The most cited authors: A total of 7,244 authors were identified in the study. Among them, 612 authors contributed to at least two publications, 201 were associated with three publications, 89 were credited with four publications, and a select group of 51 authors had a minimum of five publications. Among these 51 authors, only those with a minimum of 10,000 citations were included in the rigorous analysis, totaling 37 authors. All 37 publications authored by these individuals were interconnected. The analysis revealed the presence of seven distinct clusters, with 257 links and a TLS of 1,011 (Figure 10). The size of frames and fonts represents the number of citations. An item's color represents the authors' average citation and ranges from blue (lowest score) to green to yellow (highest score).

The Most Cited Countries

**Figure 11:**
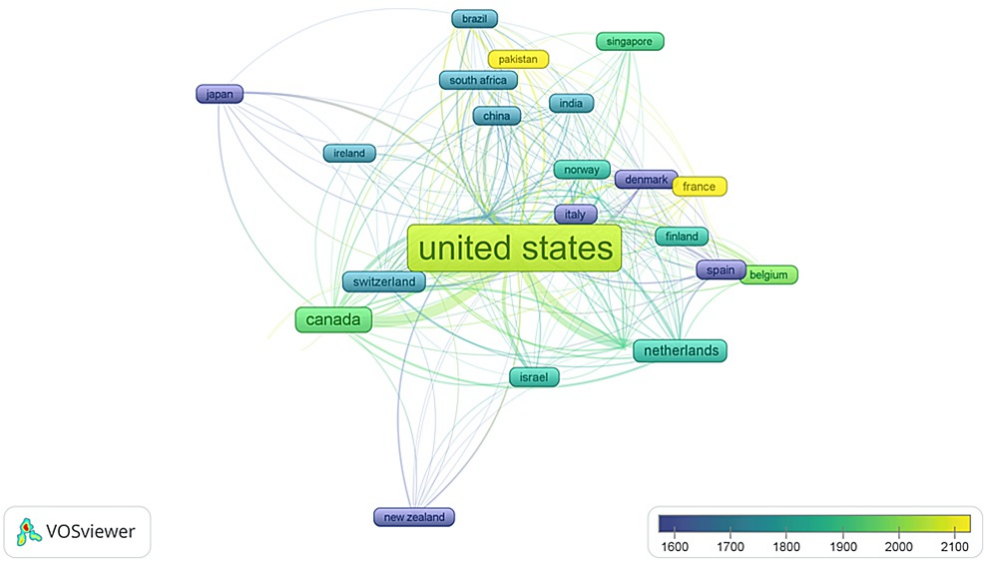
Overlay Visualization of the Most Cited Countries. The size of the frames and fonts represents the number of citations (weight-citation). Lines represent the links between the countries. The color of an item represents the average citation of the countries and ranges from blue (lowest score) to green to yellow (highest score). Image Credit: Namrata Dagli

The study employed specific criteria for analysis, setting a minimum threshold of 10,000 citations and at least five documents per country. Out of the 1,237 countries identified, only 27 nations met these criteria. Additionally, 71 countries had a minimum of two publications, 49 countries had three publications, 38 countries had four publications, and 33 countries had five publications. The visual representation (Figure 11) comprised 27 items, six distinct clusters, 256 interconnections, and a TLS of 3,993. Different colors in the graph denoted varying ranges of average citations. The analysis revealed that the USA exhibited the highest average citation in the published research papers.

The Most Cited Universities

**Figure 12:**
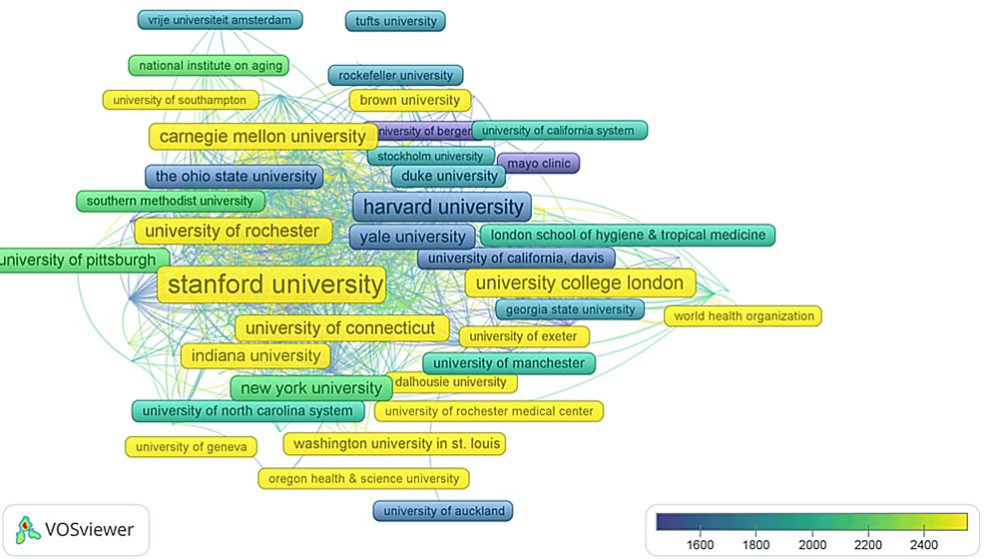
Overlay Visualization of the Most Cited Universities. The size of frames and fonts represents the number of citations (weight-citation). Lines represent the links between the universities. The color of an item represents the average citation of the item and ranges from blue (lowest score) to green to yellow (highest score). Image Credit: Namrata Dagli

A total of 2,854 universities were identified in the study. Among them, 533 universities were found to have published a minimum of two documents, 328 universities had a minimum of three publications, and 250 universities had at least four publications. Furthermore, 201 universities were observed to have published five documents or more. From this subset of 201 universities, 157 universities with a minimum of 10,000 publications were meticulously selected for further analysis. Using VOSviewer software, we calculated the total strength of citation links between these 157 universities and others. Through this analysis, universities with the highest total linkages were identified. Remarkably, the study revealed a comprehensive network connecting 156 countries. The overlay visualization unveiled intriguing patterns, encompassing 156 distinct elements, forming seven clusters. Three thousand seven hundred fifty-five links were observed in this complex network, illustrating the intricate interconnections among the universities. Notably, the TLS in this network amounted to 9,662, indicating the robustness of the relationships among the universities involved in the study (Figure 12).

The Most Cited Sources

**Figure 13:**
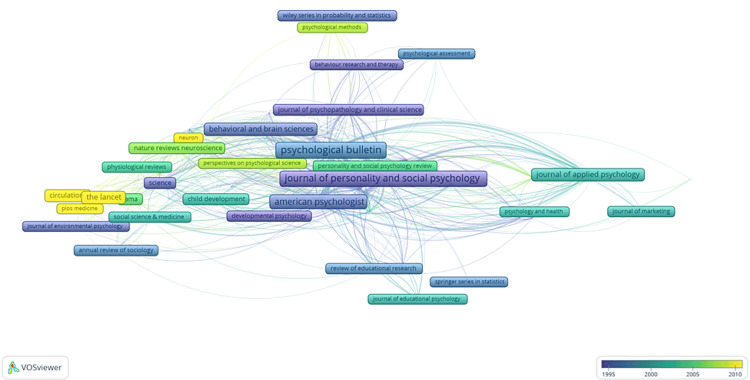
Overlay Visualization of the Most Cited Sources. The size of frames and fonts represents the number of documents (weight-documents). Lines represent the links between the items. Image Credit: Namrata Dagli

A total of 1,182 sources were identified in the study, of which 257 sources published at least two documents, and only 89 sources met the criterion of publishing a minimum of five papers. The citation threshold was set at 10,000 per source, leading to 74 sources meeting this criterion. Among these 74 sources, 72 were found to be interconnected. The overlay visualization (Figure 13) encompassed 72 items, nine distinct clusters, 565 links, and a TLS of 3,477. Notably, the yellow areas in the graph indicate sources with more recently cited research papers.

Discussion

This research paper discusses the fluctuating pattern of research paper publication over the last years, with a significant drop in 2020-2021 and a notable increase in the following year. The University of Oslo stands out as a relevant institution, and most research papers on academic stress are single-country publications. Collaboration between the Netherlands, the USA, and Australia is higher compared to other countries.

The keyword analysis suggests that a considerable focus in academic stress research has been on anxiety, quality of life, the repercussions of the COVID-19 pandemic, and the prevalence of stress experienced by medical and university students. In addition, the analysis revealed keywords linked with psychological stress, including suicidal ideation, anxiety, depressive disorder, sleep-wake disorder, obesity, oxidative stress, and hydrocortisone. Oxidative stress is, in turn, found to be linked with neoplasm. Other studies also found a link between psychological stress and suicidal ideation. They concluded that elevated academic stress and chronic stress intensify suicidal ideation, while higher resilience and good family cohesion mitigate the inclination towards suicidal thoughts stemming from stress [8,13]. A study by Steare et al. concluded that academic pressure is associated with mental health problems in adolescence [14]. A survey by Alhamed demonstrated a relationship between academic stress, sleep quality, and depressive symptoms [15]. Many studies have confirmed the relationship between psychological and oxidative stress [16-18].

According to the analysis of trends topic in our study, from 2001 to 2010, academic stress research explored various factors, shifting to nuanced areas such as psychological testing and socioeconomic determinants from 2010 to 2020. Between 2019 and 2021, there was a focus on internet addiction disorder. The thematic analysis shows the most pertinent and well-developed motor themes: university, students, psychological adaptation, psychological stress, and mental health. Additionally, emerging themes identified are oxidative stress and risk factors. Citation analysis highlights top authors, countries, universities, and sources, offering an overview of academic stress research trends.

To our knowledge, no bibliometric study has been published on academic stress. However, few bibliometric studies related to the mental health of the students have been published. The bibliometric analysis of 402 documents related to international students' mental health by Cao et al. [19] reveals an average annual growth rate of 9.31% in research on this topic. These documents are distributed across 46 countries, with a significant contribution from developed Anglo-Saxon countries such as the USA, Australia, and the UK. The analysis identifies six significant clusters of research topics within the literature: community, mental health problems, mental health service, linguistic problems, mental acculturation, and social acculturation. These clusters represent distinct schools of thought focusing on various aspects of international students' mental health, such as community-related issues, mental health problems, linguistic challenges, and social and cultural adaptation difficulties. The study's findings suggest the need for a specialized focus on international students' mental health research, offering valuable insights for future scholars and policymakers in addressing this critical issue. This study also highlights the evolution of research topics, emphasizing contemporary problems such as COVID-19 and internet and social media use, which is similar to the findings of our analysis [19].

The study by Hernández-Torrano et al. [20] analyzes the growth trajectory of publications and citations in the field of mental health and well-being in university students over the past 45 years, identifying three stages of development: an emergence stage (1975-2000), a fermentation stage (2000-2010), and a take-off stage (2010-2020). The research is primarily disseminated through psychology and psychiatry journals, with the USA being the leading country in this field. Research collaboration remains relatively scarce and mainly occurs within national borders or between culturally and geographically proximate countries. The field is interdisciplinary, with contributions from behavioral and biomedical sciences. Topical foci in the literature include positive mental health, mental disorders, substance abuse, college counseling, mental illness stigma, stress, and mental health measurement. The study highlights the need for more research from diverse regions, increased collaboration, and a broader perspective from the social sciences and humanities to enrich the field's development [20].

Another bibliometric analysis suggests that research on graduate students' mental health and well-being has grown steadily from 2012 to 2021, with a significant increase in the last two years, likely influenced by the COVID-19 pandemic. The top publishing countries are the UK, the USA, Australia, Canada, and Spain, with developed nations having a more substantial publication presence. Most research articles in this field come from universities, with European institutions leading in productivity. The research focuses on various aspects, including the general perspective of mental health in higher education, interventions, risk factors, psychological experiences, and the impact of epidemics such as COVID-19. The key findings emphasize the importance of social support, the gender gap in mental health, and the need for interventions to reduce mental distress among graduate students. It also highlights the potential benefits of stress management, meditation, yoga, mentorship programs, and extracurricular activities. Top-cited publications in the field come from journals such as the International Journal of Doctoral Studies and the International Journal of Environmental Research, which indicate research trends and influential sources [21].

While there has been limited bibliometric analysis on academic stress, numerous reviews and questionnaire studies have identified various stressors. According to Chowdhury et al. [22], critical stressors among undergraduate medical students include dissatisfaction with class lectures, the extensive syllabus, a lack of faculty guidance, and peer competition. Another study emphasized that the lack of adjustment to the university academic environment significantly stresses both male and female students [23]. Additionally, research on high school students revealed that high perceived social support positively impacted psychological well-being. Moderate academic stress was associated with higher well-being. Gender disparities were noted, with females experiencing more depression and social dysfunction. Students from high socioeconomic backgrounds reported lower anxiety, social phobia, academic frustrations, self-imposition, and academic pressures compared to middle and low-socioeconomic-status groups [24]. These studies underscore the diverse nature of academic stressors across different educational levels and types. Additionally, the scarcity of research on primary school students highlights a gap in the existing literature.

**Figure 14:**
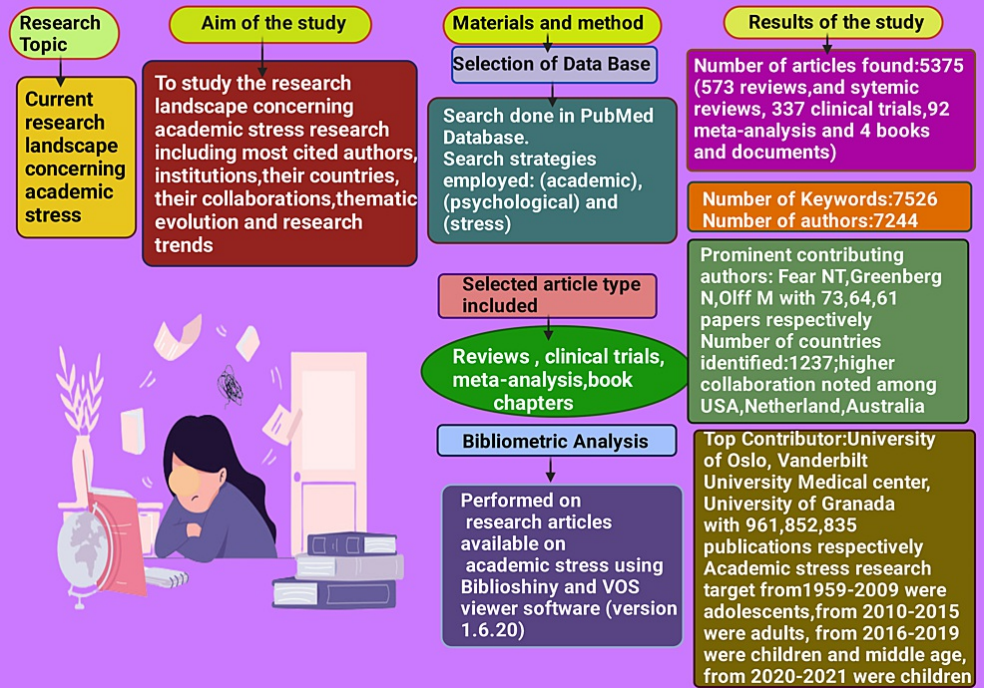
The Dominant Findings of This Paper. Notes: This figure has been drawn with the premium version of BioRender (https://biorender.com/ accessed on 30th December 2023) with the Agreement License Number VD26HQ85AX. Image Credit: Rahnuma Ahmad

Our study employed automated tools to screen articles, mitigating subjective bias and enabling large-scale data analysis. Two databases are considered for bibliometric analysis - PubMed database was considered for the study of leading authors, universities, keywords, and themes. In contrast, citation analysis was performed using the data exported from the Dimension database due to the lack of support for this analysis in the PubMed database. Despite its advantages, the study had its limitations. Firstly, it is impossible to merge the data from the two databases, so the data from these databases were analyzed separately. Secondly, analyzing each paper individually and checking their relevancy was not feasible given the vast number of articles included. Thirdly, due to the limitations of our study design, we could not pinpoint specific academic stressors. More systematic reviews are needed to identify academic stressors to comprehensively address these issues, allowing targeted interventions to alleviate them. Additionally, further in-depth investigation is required to explore the associations between psychological stress and several related terms, including suicidal ideation, anxiety, depressive disorder, sleep-wake disorder, obesity, oxidative stress, hydrocortisone, and internet addiction disorder. Nevertheless, this study is a valuable resource for identifying research trends, thematic evolution, leading researchers, and organizations, offering valuable insights for new researchers in academic stress. The findings of this paper are depicted in Figure 14.

Future Study Recommendation

To alleviate academic stress, institutions can implement stress management programs, enhance mental health support services, incorporate mindfulness practices, review assessment methods, encourage open communication, promote healthy lifestyle habits, facilitate peer support networks, educate on stress management techniques, address socioeconomic factors, provide academic support services, and continuously evaluate and adapt policies to create a supportive and balanced learning environment. The studies on primary school students are very scarce. Therefore, more research should be planned to evaluate the impact of academic stress on their mental health. The keyword co-occurrence analysis revealed a connection between stress and suicidal ideation, as well as other psychological disorders. This finding underscores the need for further investigation to better understand these associations and develop preventive measures against the negative impacts of stress.

## Conclusions

Analyzing research paper publication trends on academic stress reveals a consistent upward trajectory over the years marked by fluctuations. The University of OSLO emerges as the most relevant institution based on the number of publications in this research domain. While most papers are single-country publications, collaboration frequency between the Netherlands, the USA, and Australia stands out. The predominant keywords suggest research focus on anxiety, quality of life, and stress prevalence among medical and university students, particularly emphasizing mental health and the impact of the COVID-19 pandemic. The co-occurrence analysis identifies keywords linked with psychological stress, including suicidal ideation, anxiety, and many other psychological disorders. Thematic evolution indicates a shift from factors such as work schedules to nuanced areas like psychological testing and socioeconomic determinants, with a recent emphasis on internet addiction disorder research. The thematic map categorized the themes based on degree of development and relevancy. The emerging themes identified are oxidative stress and risk factors. The study underscores a scarcity of research on primary school students. Citation analysis unveils the most cited authors, countries, universities, and sources. This bibliometric analysis provides valuable insights into the evolving landscape of academic stress research, identifying key trends, collaborations, and thematic shifts that shape the scholarly discourse in this critical field.

## References

[1] Barbayannis G, Bandari M, Zheng X, Baquerizo H, Pecor KW, Ming X (2022). Academic stress and mental well-being in college students: correlations, affected groups, and COVID-19. Front Psychol.

[2] Deng Y, Cherian J, Khan NU (2022). Family and academic stress and their impact on students’ depression level and academic performance. Front Psychiatry.

[3] Karyotaki E, Cuijpers P, Albor Y (2020). Sources of stress and their associations with mental disorders among college students: results of the World Health Organization World Mental Health Surveys International College Student initiative. Front Psychol.

[4] Brand HS, Schoonheim-Klein M (2009). Is the OSCE more stressful? Examination anxiety and its consequences in different assessment methods in dental education. Eur J Dent Educ.

[5] Huang N, Qiu S, Alizadeh A, Wu H (2020). How incivility and academic stress influence psychological health among college students: the moderating role of gratitude. Int J Environ Res Public Health.

[6] Ignacchiti MD, Sesti-Costa R, Marchi LF, Chedraoui-Silva S, Mantovani B (2011). Effect of academic psychological stress in post-graduate students: the modulatory role of cortisol on superoxide release by neutrophils. Stress.

[7] Struthers CW, Perry RP, Menec VH (2000). An examination of the relationship among academic stress, coping, motivation, and performance in college. Res High Educ.

[8] Okechukwu FO, Ogba KT, Nwufo JI, Ogba MO, Onyekachi BN, Nwanosike CI, Onyishi AB (2022). Academic stress and suicidal ideation: moderating roles of coping style and resilience. BMC Psychiatry.

[9] Pozos-Radillo BE, de Lourdes Preciado-Serrano M, Acosta-Fernández M, de los Ángeles Aguilera-Velasco M, Delgado-García DD (2014). Academic stress as a predictor of chronic stress in university students. Psicología educativa.

[10] Page MJ, McKenzie JE, Bossuyt PM (2021). The PRISMA 2020 statement: an updated guideline for reporting systematic reviews. BMJ.

[11] Aria M, Cuccurullo C (2017). bibliometrix: an R-tool for comprehensive science mapping analysis. J Informetr.

[12] Van Eck NJ, Waltman L (2023). VOSviewer manual. https://www.vosviewer.com/documentation/Manual_VOSviewer_1.6.19.pdf.

[13] Bert F, Ferrara M, Boietti E (2022). Depression, suicidal ideation and perceived stress in Italian humanities students: a cross-sectional study. Psychol Rep.

[14] Steare T, Gutiérrez Muñoz C, Sullivan A, Lewis G (2023). The association between academic pressure and adolescent mental health problems: a systematic review. J Affect Disord.

[15] Alhamed AA (2023). The link among academic stress, sleep disturbances, depressive symptoms, academic performance, and the moderating role of resourcefulness in health professions students during COVID-19 pandemic. J Prof Nurs.

[16] Eick SM, Barrett ES, van 't Erve TJ (2018). Association between prenatal psychological stress and oxidative stress during pregnancy. Paediatr Perinat Epidemiol.

[17] Sivonová M, Zitnanová I, Hlincíková L, Skodácek I, Trebatická J, Duracková Z (2004). Oxidative stress in university students during examinations. Stress.

[18] Wang L, Muxin G, Nishida H, Shirakawa C, Sato S, Konishi T (2007). Psychological stress-induced oxidative stress as a model of sub-healthy condition and the effect of TCM. Evid Based Complement Alternat Med.

[19] Cao QT, Vuong QH, Pham HH, Luong DH, Ho MT, Hoang AD, Do MT (2021). A bibliometric review of research on international students’ mental health: science mapping of the literature from 1957 to 2020. Eur J Investig Health Psychol Educ.

[20] Hernández-Torrano D, Ibrayeva L, Sparks J (2020). Mental health and well-being of university students: a bibliometric mapping of the literature. Front Psychol.

[21] Okoro C, Owojori OM, Umeokafor N (2022). The developmental trajectory of a decade of research on mental health and well-being amongst graduate students: a bibliometric analysis. Int J Environ Res Public Health.

[22] Chowdhury R, Mukherjee A, Mitra K, Naskar S, Karmakar PR, Lahiri SK (2017). Perceived psychological stress among undergraduate medical students: role of academic factors. Indian J Public Health.

[23] Verger P, Combes JB, Kovess-Masfety V, Choquet M, Guagliardo V, Rouillon F, Peretti-Wattel P (2009). Psychological distress in first year university students: socioeconomic and academic stressors, mastery and social support in young men and women. Soc Psychiatry Psychiatr Epidemiol.

[24] Glozah FN (2013). Effects of academic stress and perceived social support on the psychological well-being of adolescents in Ghana. Open J Med Psychol.

